# Oncolytic adenovirus encoding decorin and CD40 ligand inhibits tumor growth and liver metastasis via immune activation in murine colorectal tumor model

**DOI:** 10.1186/s43556-024-00202-1

**Published:** 2024-09-22

**Authors:** Yejing Rong, Yingjun Ning, Jianping Zhu, Pei Feng, Weixin Zhu, Xin Zhao, Zi Xiong, Chunyan Ruan, Jiachang Jin, Hua Wang, Ting Cai, Shun Zhang, Yuefeng Yang

**Affiliations:** 1 Department of Experimental Medical Science, Ningbo No.2 Hospital, Ningbo, 315010 China; 2 Department of Pharmacy, Sir Run Run Shaw Hospital, School of Medicine, Zhejiang University, Hangzhou, 310016 China; 3 Ningbo Qianyang Talent Service Co., Ltd, Ningbo, 315020 China; 4 Guoke Ningbo Life Science and Health Industry Research Institute, Ningbo, 315032 China; 5 Jiangbei Center For Disease Control and Prevention Ningbo, Ningbo, 315020 China; 6 Department of Experimental Haematology, Beijing Institute of Radiation Medicine, 27 Taiping Road, Beijing, 100850 China

**Keywords:** Colorectal cancer, Immune therapy, Oncolytic adenovirus, Mouse decorin, CD40 Ligand

## Abstract

Colorectal cancer (CRC) is the second common cause of cancer mortality worldwide, and it still lacks effective approaches for relapsed and metastatic CRC. Recently, oncolytic virus has been emerged as a promising immune therapeutic strategy. In this study, we develop a novel oncolytic adenovirus, rAd.mDCN.mCD40L, which drive oncolytic activity by telomerase reverse transcriptase promoter (TERTp). rAd.mDCN.mCD40L expressed both mouse genes of decorin (mDCN) and CD40 ligand (mCD40L), and produced effective cytotoxicity in both human and mouse CRC cells. Moreover, oncolytic adenovirus mediated mDCN over-expression inhibited Met expression in vitro. In CT26 subcutaneous tumor model, intratumorally delivery of oncolytic adenoviruses could inhibit tumor growth and liver metastasis, while mDCN and/or mCD40L armed oncolytic adenoviruses produced much more impressive responses. No obvious toxicity was detected in lung, liver and spleen. Moreover, mDCN and/or mCD40L armed oncolytic adenoviruses altered the immune state to activate anti-tumor responses, including increasing CD8^+^ T effector cells and CD4^+^ memory T cells, reducing MDSCs and Tregs in peripheral blood. Furthermore, mDCN and/or mCD40L armed oncolytic adenoviruses mediated mDCN and/or mCD40L expression in tumors, and up-regulated Th1 cytokines and reduced Th2 cytokines in tumors, which will be benefit for remodeling tumor microenvironment. Importantly, rAd.mDCN.mCD40L and rAd.mCD40L prevented tumor liver metastasis much more effectively than rAd.Null and rAd.mDCN. Therefore, rAd.mDCN.mCD40L and rAd.mCD40L are promising approaches for CRC therapy.

## Introduction

 Colorectal cancer (CRC) is the second common cause of cancer mortality worldwide. There are more than 935,000 dead cases in 2020 according to the statistics by *Global Cancer Observatory database (*https://gco.iarc.fr/*).* Recurrence and distant metastasis are two major obstacles to improve the overall survival of CRC. It has been reported that distant metastasis could be detected in 20% of newly diagnosed cases [1]. Moreover, about 40% of the patients with localized diseased finally recurred after conventional therapy [2]. Therefore, it is urgent to develop much more effective therapeutic strategies to treat advanced, recurrent and metastatic CRC.

Immunotherapy has been emerged as promising strategies for cancer therapy. In recent years, immune checkpoint inhibitors, cytotoxic T-lymphocyte antigen 4 (CTLA-4) antibody and programmed death receptor 1 (PD-1) antibody has been widely used in clinic. Moreover, immune checkpoint on NK activation, CD94-NKG2A, might be a potential target for immune therapy [3]. Oncolytic virus has been emerged as a promising approach for tumor immunotherapy, and numerous oncolytic viruses has been evaluated in clinical trials [4–7]. Talimogene laherparepvec, type I oncolytic herpes simplex virus (HSV-I) expressing granulocyte macrophage colony-stimulating factor (GM-CSF), has been approved by the U.S. Food and Drug Administration (FDA) in 2015 [8–10]. Oncolytic adenovirus is one of most common oncolytic viruses, and the safety and anti-tumor effects have been validated in various clinical studies [11–15]. Our previous studies have reported that oncolytic adenovirus expressing decorin, a natural inhibitor of transforming growth factor β (TGFβ) signaling, produced obvious anti-tumor responses in various animal models [16–19]. Moreover, we also found that combining decorin and GM-CSF could slightly enhance the anti-tumor effects in CT26 model [18].

CD40-CD40L, an important costimulatory signaling pathway, participates in the activation of both humoral and cellular immunity. Both CD40 specific agonist antibody and recombinant soluble CD40 Ligand (CD40L) protein can effectively activate anti-tumor immune response in pre-clinical animal models [20–22]. However, agonist antibody and recombinant proteins could be not accurately delivered to tumor sites, which might cause serious side effects [23]. Oncolytic adenovirus mediated gene transfer could limit the protein expression at tumor lesions to improve the safety. A dual-targeting oncolytic adenovirus Ad5/3-hTERT-CD40L (CGTG-401) has achieved encouraging effects in clinical studies [24], while an oncolytic adenovirus carrying the fusion protein of tumor antigen and CD40L also effectively activated dendritic cells (DC) and inhibited the growth of prostate cancer [25].

In short, TGF-β negatively regulate anti-tumor responses in tumor microenvironment, while CD40-CD40L could activate various immune cells via enhancing co-stimulation signaling. In this study, we hypothesized that oncolytic adenovirus could lyse tumor cells to release tumor specific antigens and inhibit TGF-β signaling to remodeling tumor microenvironment, and then activated immune cells by CD40-CD40L signaling. We created an oncolytic adenoviruses, rAd.mDCN.mCD40L co-expressing murine decorin (mDCN), a natural inhibitor of TGFβ signaling and murine CD40L (mCD40L). rAd.mDCN.mCD40L could produce significant cytotoxicity in vitro. Oncolytic adenoviruses expressed mouse mDCN and/or mCD40L obviously inhibited both tumor growth and liver metastasis in murine CT26 colorectal tumor model. Moreover, rAd.mDCN.mCD40L and rAd.mCD40L produced much more impressive inhibitory effects on tumor metastasis. Unexpected, the synergistic effect between mDCN and mCD40L were not shown, and should be further evaluated.

## Results

### rAd.mDCN.mCD40L induces cytotoxicity and expresses decorin and CD40L in the colon cancer cell lines

**Fig. 1:**
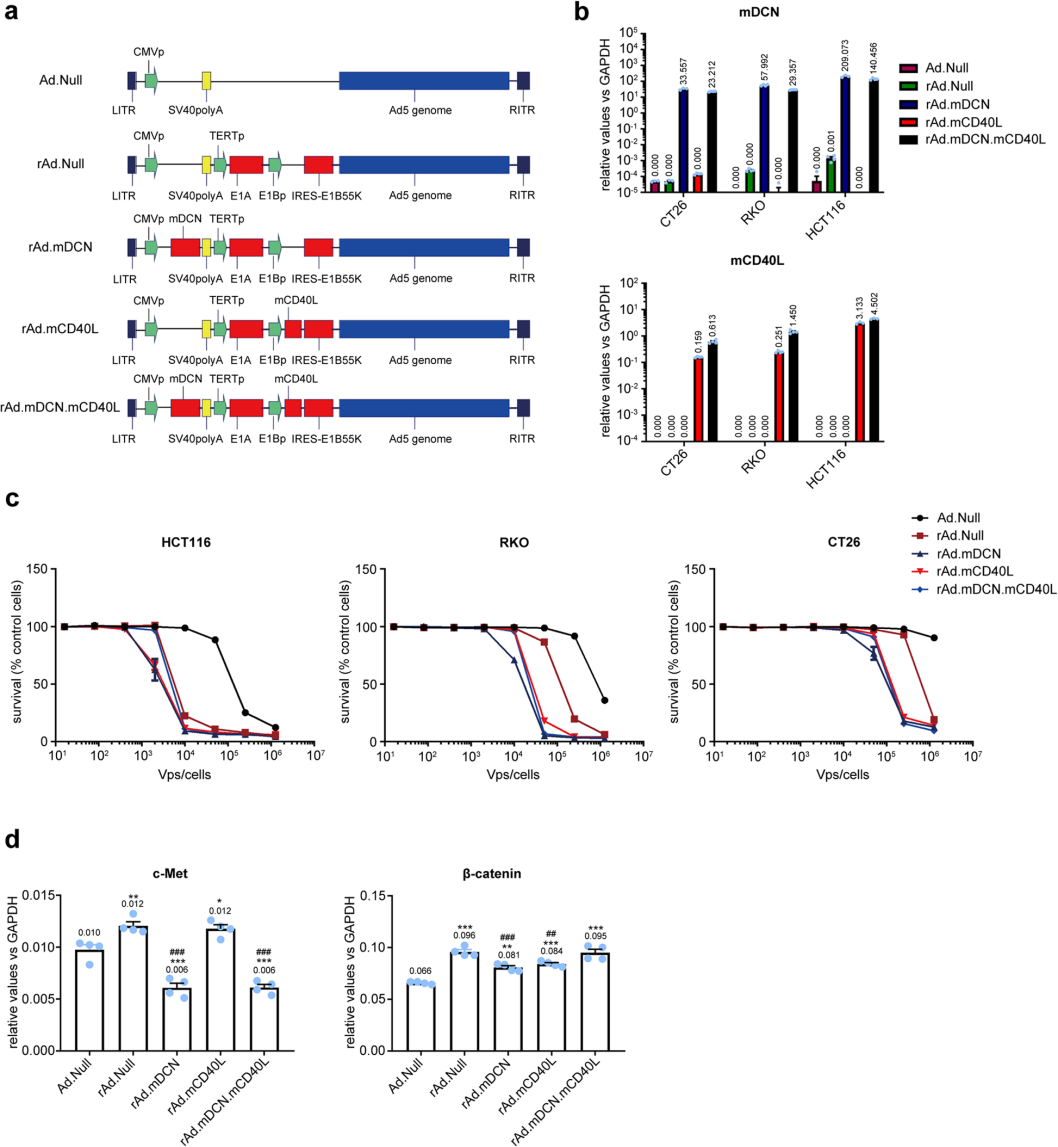
The construction and in vitro evaluation of oncolytic adenovirus, rAd.mDCN.mCD40L. **a** The scheme of replication-deficiency adenovirus, Ad.Null, and oncolytic adenoviruses, rAd.Null, rAd.mDCN, rAd.mCD40L and rAd.mDCN.mCD40L. **b** Decorin and CD40L mRNA expression in colon cancer cells (CRCs), including HCT116, RKO, and CT26 cells. The CRC cells were infected by Ad.Null, rAd.Null, rAd.mDCN, rAd.mCD40L and rAd.mDCN.mCD40L at multiplicity of infection (MOI) of 2.0 × 10^4^ vp/cell. Twenty-four hours after infection, cells were harvested and the mRNA expression of decorin and CD40L were detected by real-time reverse transcription polymerase chain reaction (RT-PCR). **c** Oncolytic adenoviral-induced cytotoxicity in CRCs. CRCs were infected with various viruses at serial viral doses, ranging from 80 to 1.25 × 10^6^ vp/cell. Seven-days after infections, the cell survival was analyzed by sulforhodamine B (SRB) staining. **d** c-Met and β-catenin mRNA expression in CT26 cells. CRCs were infected, collected and the expression of c-Met and β-catenin were analyzed by real-time RT-PCR as described above. Data in (**d**) are shown as mean ± SEM; *p < 0.05, **p < 0.01, and ***p < 0.001 compared with Ad.Null-infected cells. ##*p* < 0.01, and ###*p* < 0.001 compared with rAd.Null-infected cells

Both decorin, a natural inhibitor of TGF-β signaling, and CD40L, one of the strongest inducers of Th1 responses, are potential target for tumor therapy [26–28]. In this study, we constructed an oncolytic adenovirus expressed mouse decorin (mDCN) and CD40L (mCD40L), the viral replication was regulated by hTERT Promoter (Fig. 1a). As expected, rAd.mDCN.mCD40L expressed both *mDCN* and *mCD40L* at high level in CRC cells (Fig. 1b). In general, oncolytic adenoviruses produced obvious dose-dependent cytotoxicity in human CRC cells, HCT116 and RKO, as well as in the mouse CRC cell line, CT26. HCT116 is most sensitive cell line to oncolytic adenovirus, while CT26 is the most resistant cell line (Fig. 1c). The lower cytotoxicity in CT26 cells might be attributed to that human derived oncolytic adenovirus could not replicate in mouse cells [18]. Two pivotal target genes of decorin, *c-met* and *β-catenin* were analyzed in CT26 cells after viral transduction. We found that mDCN over-expression significantly reduced level of *c-met*, but not *β-catenin* in CT26 cells (Fig. 1d). These results suggested that rAd.mDCN.mCD40L mediated over-expression of *mDCN* and *mCD40L*, induced cytotoxicity in CRC cells, as well as inhibited tumor growth and metastasis associated genes.

### rAd.mDCN.mCD40L inhibits tumor growth in CT26 subcutaneous tumor model

**Fig. 2:**
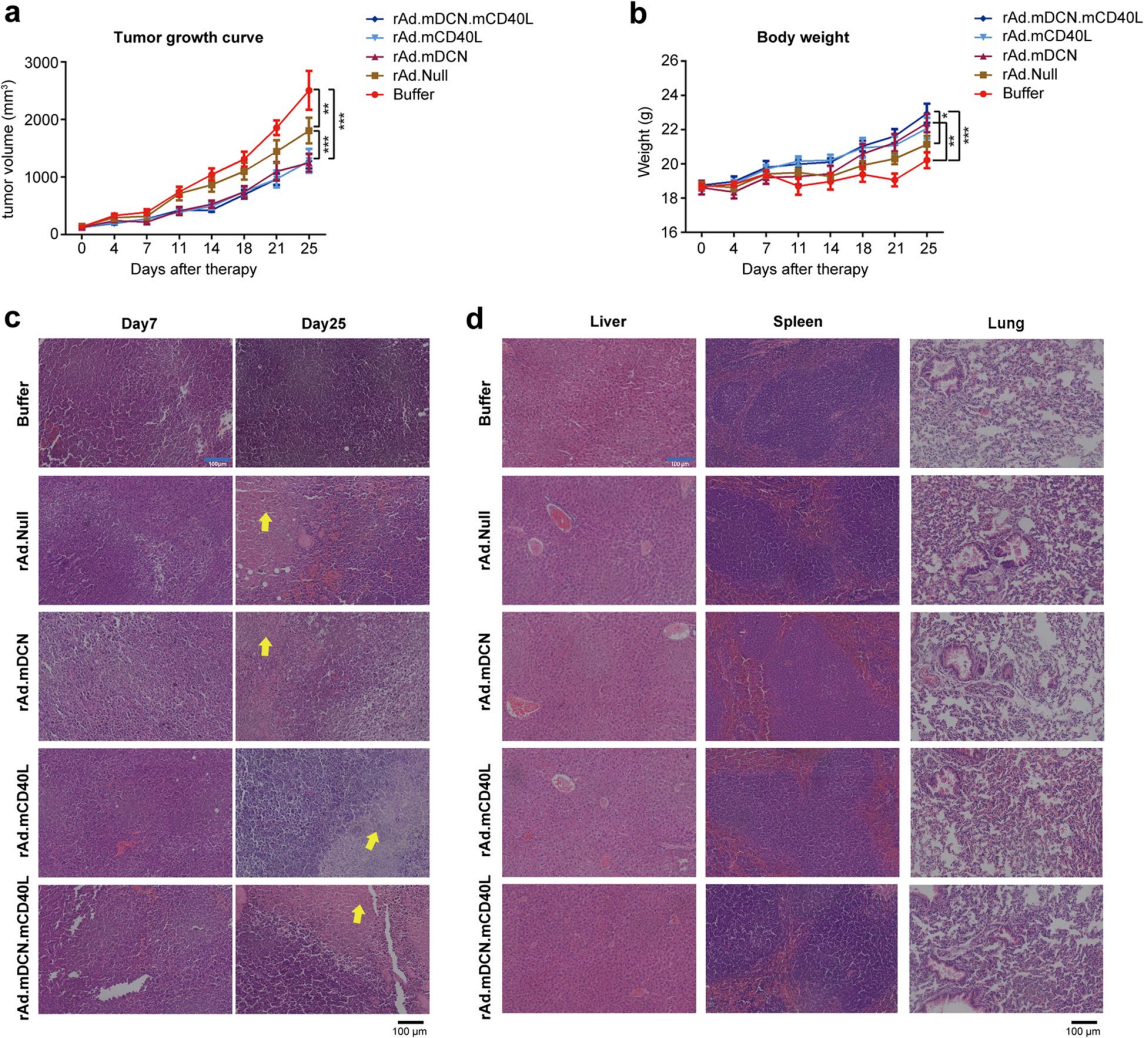
rAd.mDCN.mCD40L produces antitumor responses in CT26 subcutaneous tumor model. To establish CT26 subcutaneous tumor model, 2 × 10^6^ CT26 cells were injected subcutaneously into BALB/c mice. Fourteen days after the cell injection, tumor-bearing mice were divided into 5 groups without statistical difference in tumor volume (*n* = 20/group). 2.5×10^10^ VPs rAd.mDCN.mCD40L, rAd.mDCN, rAd.mCD40L, rAd.Null, or PBS was administrated intratumorally on day 0. A repeated injection was conducted on day 3. Tumor volumes and body weight were monitored twice a week and are presented in (**a**) and (**b**). The histopathological changes in tumor tissues (**c**), liver, spleen and lung (**d**) were analyzed by hematoxylin-eosin (H&E) staining both on day 7 and 25 (**c**). Data in (**a**) and (**b**) are shown as mean ± SEM. *p < 0.05, **p < 0.01, and ***p < 0.001, vs Buffer group (  )

In murine CT26 model, oncolytic adenoviruses significantly inhibited tumor growth. And, rAd.mDCN.mCD40L, rAd.mDCN, and rAd.mCD40L produced much stronger inhibitory effects than rAd.Null, indicating that both mDCN and mCD40L could enhance the anti-tumor responses of oncolytic adenovirus. On day 25, the tumor growth inhibition index in rAd.Null group, rAd.mDCN group, rAd.mCD40L group and rAd.mDCN.mCD40L group were 27.92%, 50.22%, 48.78% and 48.95%, respectively (Fig. 2a). However, no obvious elevation was detected by co-express mDCN and mCD40L. We also monitored animal body weight twice a week, which is a valuable indicator of cancer cachexia. We found that the average body weight in oncolytic adenoviruses treated groups, especially that in rAd.mDCN.mCD40L group, was higher than buffer group on day 25 (Fig. 2b). These results suggested that rAd.mDCN.mCD40L might prevent cancer cachexia. The histopathological analysis showed oncolytic adenoviruses treatments increased inflammatory infiltration in tumor tissues both on day 7 and day 25, as well as induced necrosis on day 25 (Fig. 2c). Importantly, H&E staining suggested that no obvious histopathological changes could be detected in livers, spleens and lungs after treatment with oncolytic adenoviruses on day 7, indicating that no signicant toxicity to liver, spleen and lung could be observed after intratumoally adminstration of oncolytic adenoviruses (Fig. 2d).

### rAd.mDCN.mCD40L expresses therapeutic genes and induces apoptosis in tumor tissues

**Fig. 3:**
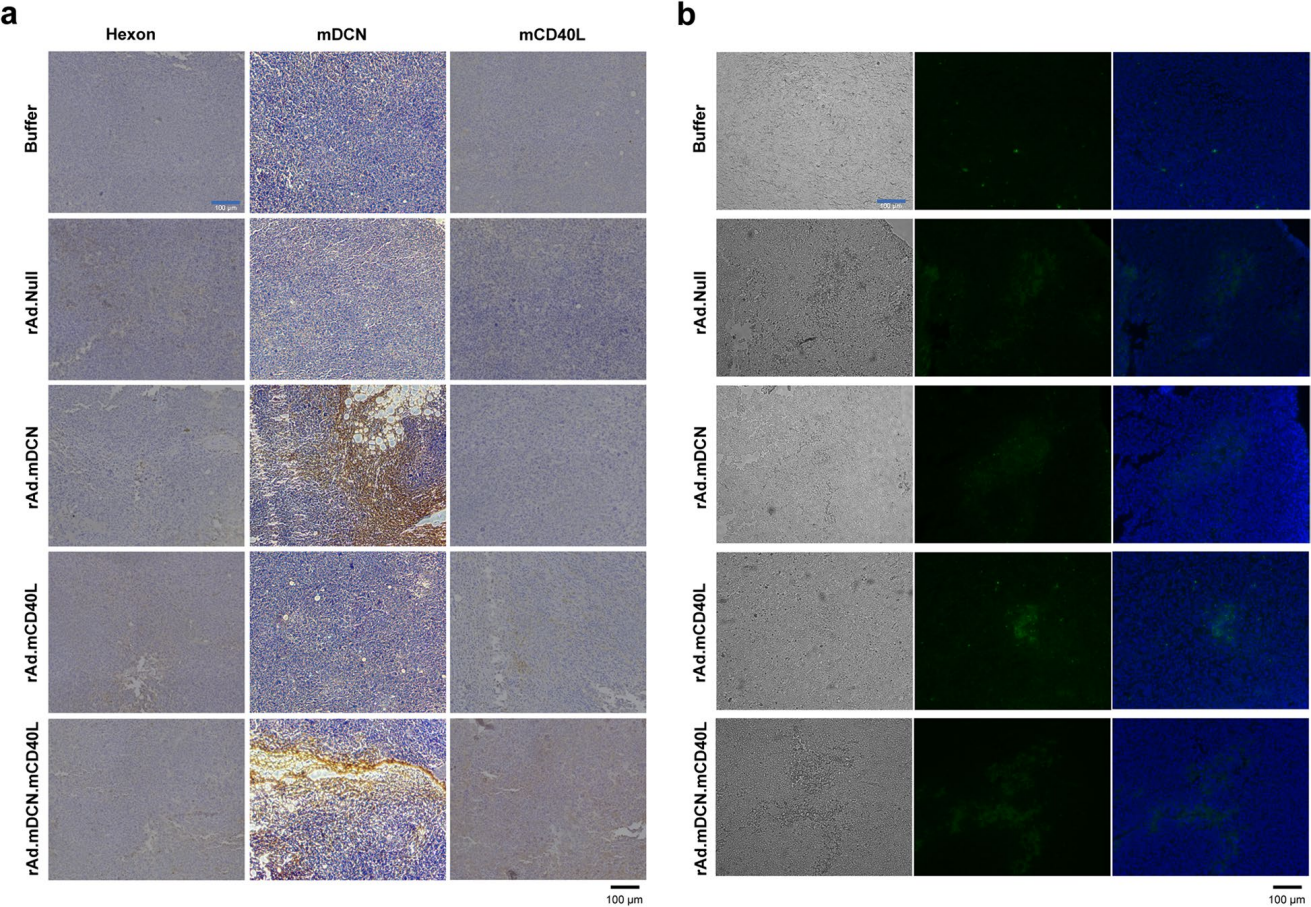
rAd.mDCN.mCD40L mediated expression of therapeutic genes and induced apoptosis/death of tumor cells in vivo. Murine CT26 subcutaneous tumor model was established and oncolytic adenoviruses were administrated as described in materials and methods. On day 7 after treatment, the mice from each group were euthanized and the tumor tissues were removed. The protein expression of hexon, mDCN and mCD40L were analyzed by immunohistochemistry (IHC) (**a**). In addition, oncolytic adenovirus-induced apoptosis was evaluated by terminal deoxynucleotidyl transferase dUTP nick end labeling (TUNEL) (**b**)

Gene modification could enhance the therapeutic effects by introducing cellular apoptosis associated genes, immune activation related genes and so on. In this study, we found that adenoviruses widely-distributed in tumor tissues by analyzing hexon protein expression via IHC, on day 7 after treatment. As expected, both rAd.mDCN and rAd.mDCN.mCD40L produced high level mDCN, while rAd.mCD40L and rAd.mDCN.mCD40L significantly increased mCD40L in tumor tissues (Fig. 3a). These data suggested that intratumoral delivery of oncolytic adenoviruses could effectively infect tumor cells and express therapeutic genes at high level.

Next, viral-induced cell apoptosis/death is an important mechanism underlying oncolytic virus mediated anti-tumor responses. Our data showed that all the oncolytic adenoviruses could induce cell apoptosis/death in the tumor lesions. However, rAd.mCD40L and rAd.mDCN.mCD40L treatments produced much more impressive effects, which indicated that mCD40L might promote the oncolytic virus induced apoptosis of tumor cells (Fig. 3b).

### rAd.mDCN.mCD40L inhibits tumor liver metastasis in CT26 subcutaneous tumor model

**Fig. 4:**
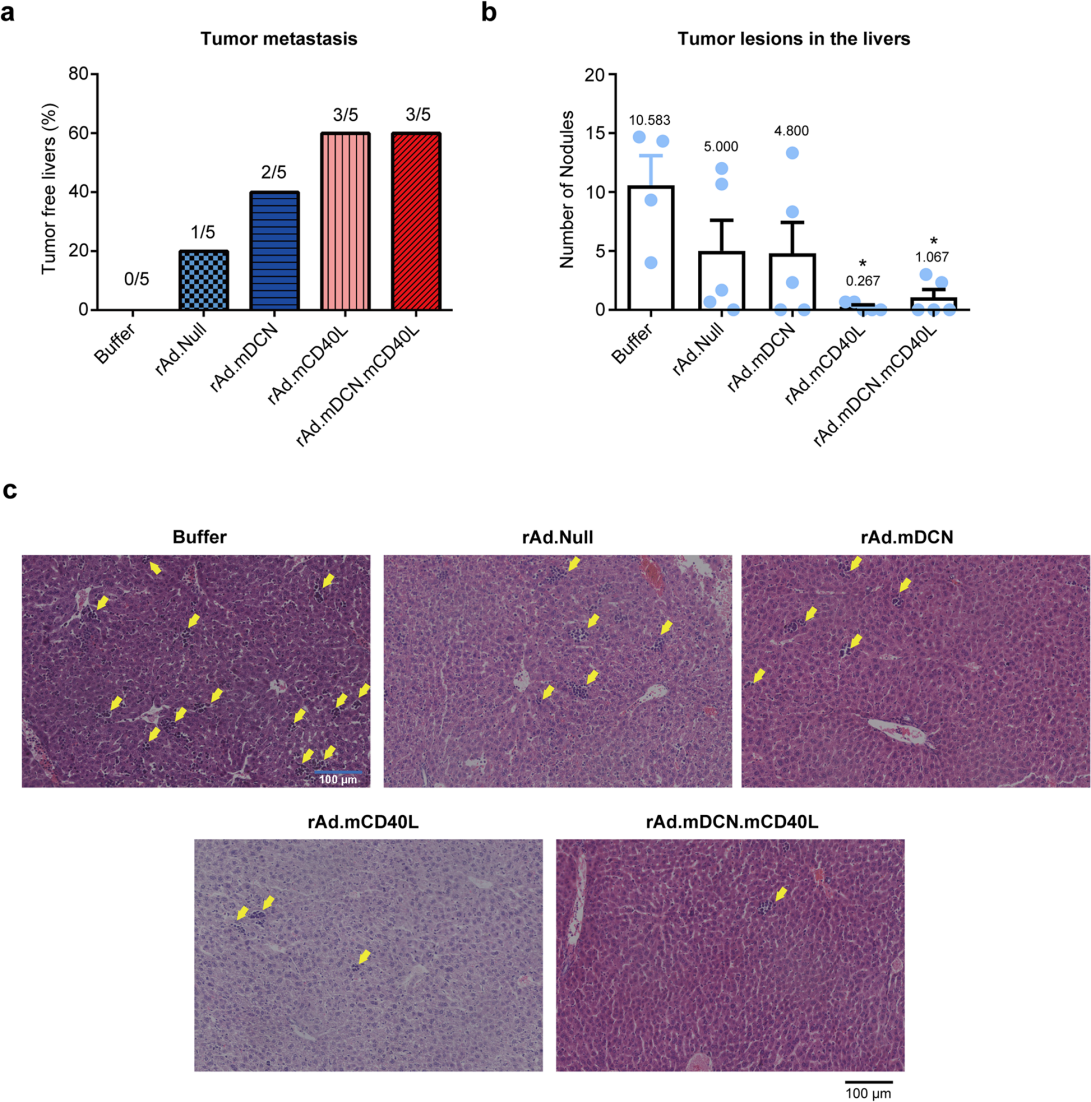
rAd.mDCN.mCD40L inhibits liver metastasis in vivo. Murine CT26 subcutaneous tumor model was established and treated with oncolytic adenoviruses as described in materials and methods. On day 25 after treatment, five mice from each group were euthanized and the livers were removed. The tumor metastatic lesions in livers were observed, and tumor-free livers were counted (**a**). Moreover, the metastatic lesions were counted, and the average metastatic lesions in each liver were calculated and presented in (**b**). And, the representative images of H&E staining were shown in (**c**). Data in (**b**) were shown as mean ± SEM. *p < 0.05, vs. Buffer group

Distant metastasis frequently occurred at the advanced stage of various tumors, and always closely associated with the poor prognosis. Liver is the most common organ for CRC distant metastasis. On day 25 after treatment, liver metastasis could be detected in all of the mice from buffer group. Generally, oncolytic adenoviruses treatments inhibited liver metastasis of murine CT26 tumor model. Moreover, rAd.mCD40L and rAd.mDCN.mCD40L inhibited tumor liver metastasis much more effectively than other oncolytic adenoviruses (Fig. 4a). The tumor nodules in livers of the tumor metastatic mice were also counted, and rAd.mCD40L and rAd.mDCN.mCD40L significantly decreased the number of nodules in liver (*p* < 0.05) (Fig. 4b and c). These results suggested that the therapeutic gene, mCD40L, might be the pivotal factor in inhibiting tumor liver metastasis.

### Potential mechanisms underlying rAd.mDCN.mCD40L mediated anti-tumor responses

**Fig. 5:**
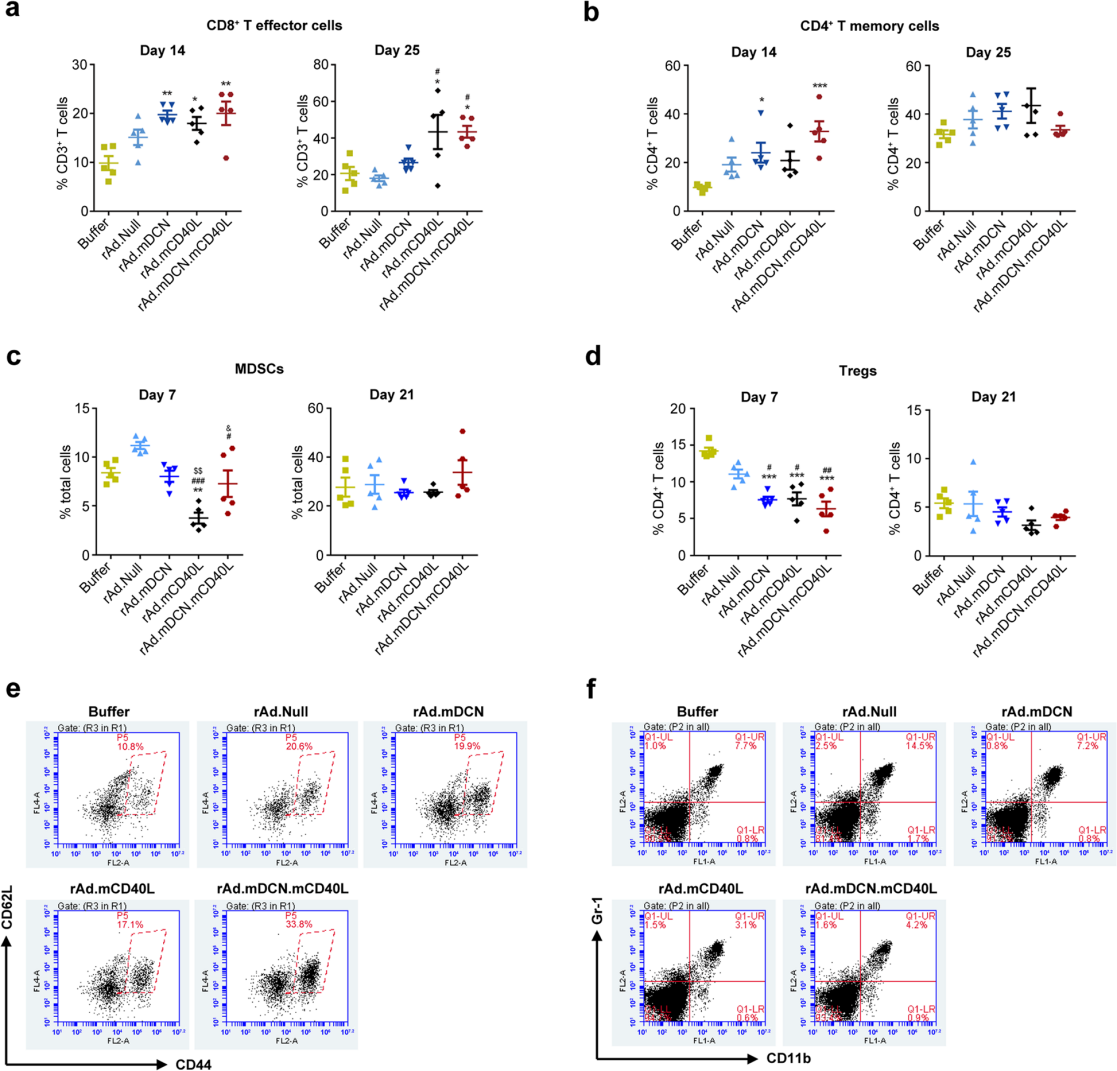
rAd.mDCN.mCD40L activates immune cells in vivo. On indicated time points, the heparin treated un-coagulated peripheral blood was collected from each group. Both on day 14 and 25, the percentage of effector T cells from CD8^+^ T lymphocytes, and memory T cells from CD4^+^ T were analyzed by flow cytometry, and were presented in (**a**) and (**b**) respectively. The representative image of analyzing CD4^+^ T memory cells on day 14 were shown in (**e**). Moreover, the percentage of myeloid-derived suppressor cells (MDSCs) and regulatory cells T cells (Tregs) were also detected by flow cytometry, and the statistical data were shown in (**c**) and (**d**) respectively. And, the representative image of MDSCs were presented in (**f**). Data in (**a**), (**b**), (**c**) and (**d**) were shown as mean ± SEM. *p < 0.05, **p < 0.01, ***p < 0.001, vs Buffer group (  ). #*p* < 0.05, vs rAd.Null group (  ). &*p* < 0.05, vs rAd.mCD40L group (  )

Cytotoxic T lymphocytes (CTLs) are the major immune effectors that can directly kill tumor cells and produce antitumor responses. Here, we found that therapeutic genes armed oncolytic adenoviruses significantly stimulated CD8^+^ effector T cells in peripheral blood on day 14. However, only rAd.mCD40L and rAd.mDCN.mCD40L obviously elevated CD8^+^ effector T cells on day 25 (Fig. 5a). We also analyzed CD4^+^ T memory cells, which are pivotal in activating both cellular immunity and humoral immunity. Our data showed that oncolytic adenoviruses increased CD4^+^ T memory cells on day 14, however statistical difference only could be detected in rAd.mDCN and rAd.mDCN.mCD40L groups. And, the percentage of CD4^+^ T memory cells out of total CD4^+^ T cells restored to normal level at day 25 after treatments (Fig. 5b and e). It has been widely demonstrated that MDSCs could be induced in cancer patient, and inhibit the activities of lymphocytes. Interestingly, we found that rAd.Null could slightly increase MDSCs in peripheral blood on day 7, while therapeutic genes armed oncolytic adenoviruses inhibited the elevation. And, the MDSCs in peripheral blood were restored to normal level on day 21 (Fig. 5c and f). MDSCs could inhibit anti-tumor immune responses via multiple mechanisms, such as inducing Tregs. In this study, we showed that oncolytic adenoviruses reduced Tregs in peripheral blood on day 7, while therapeutic genes armed oncolytic adenoviruses produced much stronger inhibitory effects. However, the percentage of Tregs in peripheral blood was recovered on day 21 (Fig. 5d). In a word, rAd.mDCN.mCD40L might induce anti-tumor immune responses via activating CD8^+^ T effector cells and CD4^+^ T memory cells, while downregulating MDSCs and Tregs.

### rAd.mDCN.mCD40L modulate Th1 and Th2 cytokines in tumors

**Fig. 6:**
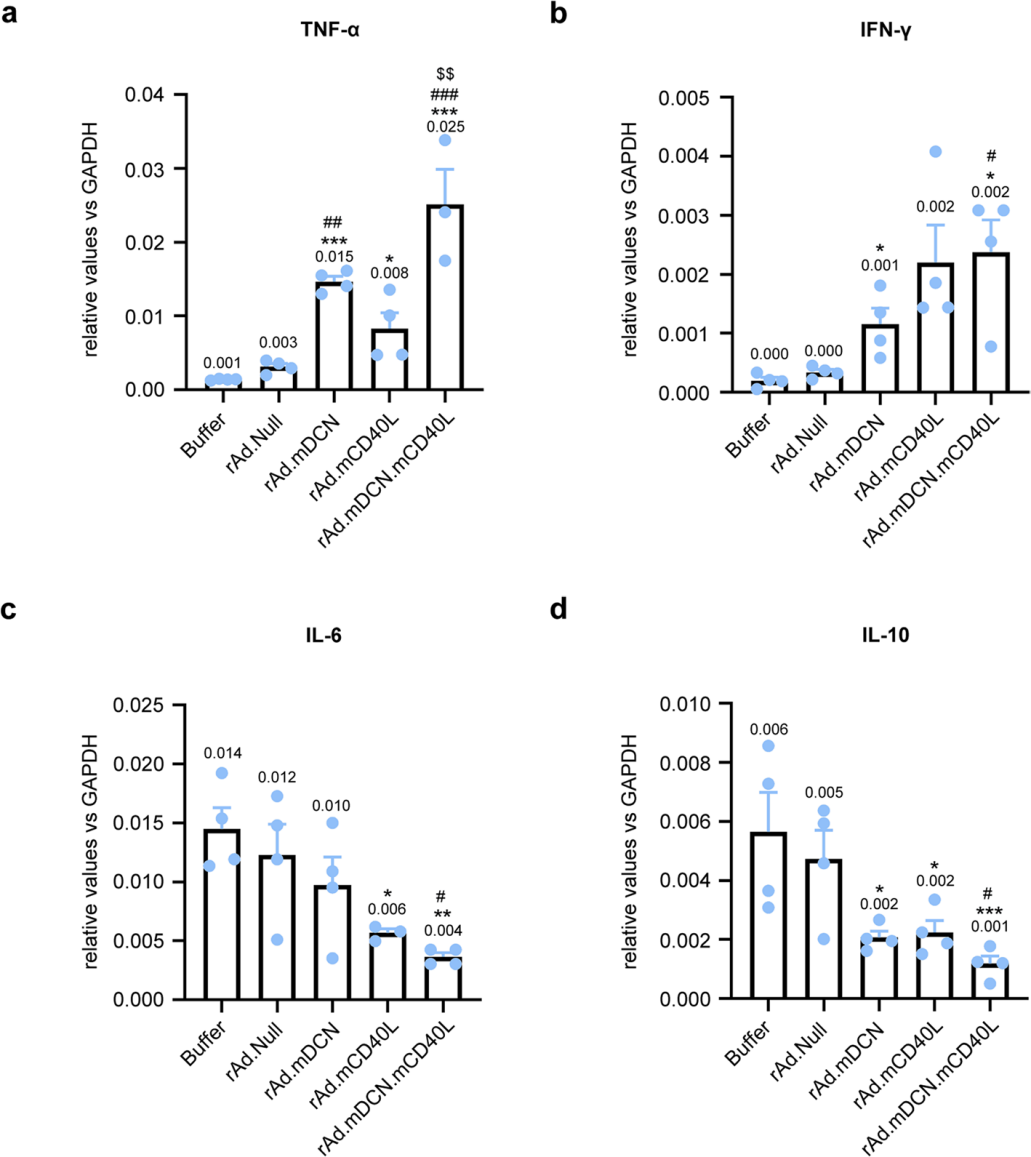
rAd.mDCN.mCD40L promoted Th1 cytokines expression while inhibited Th2 cytokines expression in tumors in CT26 subcutaneous tumor model. On day 25 after treatments, the mice from each group were euthanized and the tumor tissues were removed. The total RNA was isolated and the cDNA was synthesized as described in *Materials and Methods*. The expression of tumor necrosis factor α (*TNF-α*) (**a**), interferon γ (*IFN-γ*) (**b**), interleukin 6 (*IL-6*) (**c**) and *IL-10* (**d**) were detected by real-time reverse transcription polymerase chain reaction (RT-PCR), and the relative expression was normalized by the expression of glyceraldehyde-3-phosphate dehydrogenase (GAPDH). Data were shown as mean ± SEM. *p < 0.05, ***p < 0.001, vs. Buffer group. ##*p* < 0.01, ###*p* < 0.01, vs. rAd.Null group. $$*p* < 0.01, vs. rAd.mCD40L group

Both the Th1 cytokines, tumor necrosis factor α (*TNF-α*) and interferon γ (*IFN-γ*), and Th2 cytokines, interleukin *(IL)-6* and *IL-10* were detected in tumors on day 25 after treatments. Generally, therapeutic gene armed oncolytic adenoviruses increased expression of *TNF-α* and *IFN-γ*, while down-regulated expression of *IL-6* and *IL-10* (Fig. 6). Moreover, rAd.mDCN and rAd.mDCN.mCD40L up-regulated *TNF-α* much stronger than rAd.mCD40L. And, rAd.mCD40L and rAd.mDCN.mCD40L produced much more impressive regulatory effects on expression of *IFN-γ* and *IL-6*. These results suggested that mDCN and mCD40L might modulate anti-tumor responses via different mechanisms.

## Discussion

In the past decades, novel immune therapeutic strategies, such as immune checkpoint inhibitor, chimeric antigen receptor T cells (CAR-T) and oncolytic virus, have been emerged as promising approaches for cancer therapy [29–32]. However, there still lacks effective treatment for recurrent and metastatic CRC patients. Local immune suppressive microenvironment of solid tumors is the pivotal obstacle for immune therapy [33]. Oncolytic viruses not only directly kill tumor release, but also elicited antitumor immune responses via releasing tumor specific antigens and promoting immune cell infiltration in tumor lesions [34, 35]. The safety of serotype 5 oncolytic adenoviruses have been demonstrated in clinical trials [36]. In this study, we developed an oncolytic adenovirus expressing both mDCN and mCD40L, which produced obvious anti-tumor effects in CT26 subcutaneous tumor model.

Decorin, a well-known tumor suppressor, frequently down-regulated in tumor tissues of prostate cancer, breast cancer, as well as CRC [37–39]. Decorin can target multiple tyrosine kinase receptors, such as c-met, EGFR, and IGFR, which play important roles to support tumor growth and promoting tumor metastasis [40]. And, we have previously reported that decorin was significantly down-regulated, while Met was increased obviously in tumor tissues of CRC patients [18]. In this study, we showed that oncolytic adenoviruses expressing mDCN reduced the Met expression in CT26 cells. However, β-catenin, another important target gene of decorin and an important regulatory protein of WNT signaling pathway, was only slightly down-regulated.

It has been well-known that decorin is a natural inhibitor of TGFβ signaling, which plays an important role in regulating tumor proliferation, apoptosis, angiogenesis, epithelial-mesenchymal transition (EMT) and so on [41, 42]. TGFβ is an important component of tumor immune suppressive microenvironment, and induced immune tolerance through mainly through suppressing maturation of T helper cells, DCs and NK cells, inhibiting cytotoxicity of CD8^+^ T cells, as well as inducing M2 polarization of macrophage cells [43–46]. Both pre-clinical studies and clinical trials showed that blocking TGFβ signaling could effectively inhibit tumor growth and metastasis via activating anti-tumor immune responses [18, 19]. Therefore, TGFβ signaling pathway has been emerged as a promising target for tumor immune therapy. Moreover, combining TGFβ targeted strategies with conventional therapies, such as chemotherapy, radiation therapy and immune checkpoint inhibitors, could enhance tumor suppression effects [47, 48]. Previous studies have reported that decorin can effectively inhibit TGF-β signaling and enhance the therapeutic effect of oncolytic viruses on tumor metastasis [18, 26, 49]. Here we found that both rAd.mDCN and rAd.mDCN.mCD40L obviously inhibited tumor growth and liver metastasis. Moreover, mDCN expressed oncolytic adenoviruses increased Th1 cytokines and reduced Th2 cytokines obviously, which will be benefit for abolishing immune tolerance. Flow detection results also indicated that mDCN expressed oncolytic adenoviruses increased the proportion of CD8^+^ cells. Previous studies have found that oncolytic adenovirus can decrease the proportion of TIM-3^+^ subset of tumor-infiltrating CD8^+^ T cells, which is associated with improved survival of cancer patients [50]. This provides an idea for us to further study the mechanism of rAd.mDCN.mCD40L inhibiting the growth of colorectal cancer.

CD40-CD40L play pivotal roles in activating anti-tumor responses, and has been emerged as a potential target of tumor immune therapy. CD40 is frequently expressed on DCs, B cells, T cells, monocytes as well as several tumor cells [51]. CD40L is a type II transmembrane protein, which belongs to the tumor necrosis factor (TNF) superfamily, mainly expressed on CD4^+^ T cells. Several groups reported that CD40 agonist antibodies and recombinant soluble CD40L could activate anti-tumor immune responses both in pre-clinical studies. However, only modest antitumor activity could be detected in clinical trials due to dose-limiting toxicity [52, 53]. Delivery of CD40L by viral vectors, especially oncolytic viral vectors, could stimulate CD40 signaling and elicit strong immune responses against tumor cells and produced impressive anti-tumor effects both in pre-clinical animal models and cancer patients [54–58]. Our previous study have demonstrated that oncolytic adenovirus expressing fusion protein of CD40L and prostate specific antigen (PSA) promote the maturation of DCs and inhibit the growth of prostate cancer obviously [25]. Garofalo et al. [59] tested the local administration of a novel oncolytic adenovirus AdV-D24-ICOSL-CD40L expressing co-stimulatory molecules ICOSL and CD40L, found that its anti-cancer effect positively correlated with cytotoxic CD8^+^ tumor-infiltrating lymphocytes exerting a central role in the tumor volume control, which is similar to our results. In addition, we found that mCD40L produced impressive inhibitory effects on liver metastasis of CRC.

In this study, we hypothesized that decorin could inhibit TGF-β signaling to remodeling tumor microenvironment and CD40L further enhance the activation of immune cells. However, there lacks proper in vitro models to evaluate the synergistic effects of mDCN and mCD40L. With the development of technologies, 3D spheroid has been emerged as a effective tools, which could mimic tumor microenvironment [60, 61]. We are interested in evaluate the immune mechanisms by using 3D spheroid system in our future studies.

In addition, a growing body of evidence suggests that epigenetic modification [62], Hedgehog and Notch signaling pathway [63] and small nucleolar RNAs (snoRNAs) [64] might be excellent targets for tumor treatment. Hao et al. [65] showed that photodynamic therapy combined with immune checkpoint blockade can effectively inhibit the growth of solid tumors and improve the survival rate of tumor-bearing mice compared to a single treatment. We can develop better cancer immunotherapy methods from these aspects in future studies. Moreover, oncolytic adenovirus mediated cell lysis is also important for immune activation. However, our oncolytic adenovirus cannot replicate in murine cells. Therefore, a humanized mice model will be necessary to evaluate the anti-tumor responses of oncolytic adenoviruses, especially that expressing immune genes.

In conclusion, we successfully developed a novel oncolytic adenovirus rAd.mDCN.mCD40L, which produced effective cytotoxicity, and expressed mDCN and mCD40L in both human and mouse CRC cells. In CT26 subcutaneous tumor model, we found that oncolytic adenoviruses obviously inhibited tumor growth and liver metastasis. However, mDCN and/or mCD40L armed oncolytic adenoviruses produced much more impressive responses. Importantly, mDCN and/or mCD40L armed oncolytic adenoviruses increased CD8^+^ T effector cells and CD4^+^ memory T cells, reduced MDSCs and Tregs, as well as up-regulated Th1 cytokines and down-regulated Th2 cytokines. Our data suggested that both rAd.mDCN.mCD40L and rAd.mCD40L are promising approaches for CRC therapy. However, the synergistic effect of mDCN and mCD40L were not shown in this model and further investigation should be conducted.

## Materials and methods

### Cell lines

Human colon-cancer cell line, RKO, was purchased from National Collection of Authenticated Cell Cultures (Shanghai, China). Mouse colon-cancer cell line, CT26, human embryonic kidney cell line, HEK293, and human colon-cancer cell line, HCT116, were obtained from American Type Culture Collection (ATCC, Manassas, VA). RKO, HCT116, and CT26 cells were maintained in RPMI-1640 media (Gibco, Grand Island, NY, USA) containing 10% fetal calf serum (FCS). HEK293 cells were cultured in Dulbecco’s minimal essential medium (DMEM; Gibco) supplemented with 10% FCS.

### Adenoviruses

The E1A expression of adenoviruses, which determines the viral replication in cells, is controlled by human telomerase reverse transcriptase promoter (hTERTp). This design ensures that the virus preferentially replicates in cells with active telomerase, notably cancer cells, given that hTERT activity is commonly elevated in these cells. rAd.mDCN.mCD40L encodes both mDCN and mCD40L, which are regulated by cytomegalovirus (CMV) promoter and E1B promoter respectively. Oncolytic adenoviruses rAd.mDCN and rAd.mCD40L are designed to express mDCN and mCD40L respectively. rAd.Null, an oncolytic adenovirus without any transgene, was used as control vector in this study. The oncolytic adenoviruses were constructed and prepared by a simplified system for generating oncolytic adenovirus vector carrying one or two transgenes, as described previously [16]. Non-replication adenovirus Ad.Null were constructed by Ad-easy system, as described previously [18].

### Adenoviral-mediated cytotoxicity in CRC cells

Human CRC cells, RKO and HCT116, and mouse CRC cell, CT26 were plated into 96-well plates at a density of 1 × 10^3^ cells/well. On next day, cells were infected with gradient diluted oncolytic adenoviruses from 16 VPs/cell to 1.25 × 10^6^ VPs/cell, and the cells were continued to incubation for 7 days as described previously [18]. The cell survival was determined by the sulforhodamine B staining using uninfected cells as control.

### Adenoviral-mediated expression of mDCN, mCD40L, and genes regulated by decorin in the colon-cancer cells

CRC cells, RKO, HCT116 and CT26, were plated into six-well plates at a density of 5 × 10^5^ cells/well. The next day, cells were infected with oncolytic adenoviruses at 2.0 × 10^4^ VPs/cell. Six hours post infection, the culture media were replaced by fresh serum-free media, and continue to culture for another 24 h. The cells were collected and total RNA was extracted from cells using RNAiso reagent (Takara, Shiga, Japan) according to the manufacturer’s instructions. Single-stranded cDNA was synthesized using ReverTra Ace qPCR RT Master Mix (Toyobo Co., Ltd., Osaka, Japan). And, the gene expression was analyzed by real-time reverse transcription polymerase chain reaction (RT-PCR).

### Real-time reverse transcription polymerase chain reaction (RT-PCR)

The mRNA expression of mDCN, mCD40L, mouse Met (mMet), mouse CTNNB1 (mCTNNB1), mouse glyceraldehyde-3-phosphate dehydrogenase (GAPDH) and human GAPDH were detected by real-time RT-PCR on a SLAN-96P fluorescent quantitative PCR instrument (Hongshi Medical Technology Co, Shanghai, China) using SYBR premix Ex Taq (Perfect Real Time; TaKaRa, Shiga, Japan)). The relative mRNA expression of the target gene were normalized to mGAPDH or hGAPDH using the comparative Ct method (2^−△△Ct^ method). And, primer pairs for above mentioned genes were listed in Table 1).

**Table 1:** Oligonucleotide primers for real-time reverse transcription polymerase chain reaction (RT-PCR)

Primer	Gene	Nucleotide sequence (5′→3′)
mDCN F	mDCN	TCTCCGCAGTTGGGCAAAATGAC
mDCN R		TGGCAGAACGCACATAGACACATC
mCD40L F	mCD40L	GAAATGCAAAGAGGTGATGAGG
mCD40L R		TCAGCTGTTTCCCATTTTCAAG
mMet F	mMet	CCGTAGACTCTGGGTTGC
mMet R		ATCTGGCTTGCTTTGTGC
mCTNNB1 F	mCTNNB1	GGGTGCTATTCCACGACT
mCTNNB1 R		CCCTTCTACTATCTCCTCCAT
mGAPDH F	mGAPDH	AGGTCGGTGTGAACGGATTTG
mGAPDH R		GGGGTCGTTGATGGCAACA
hGAPDH F	hGAPDH	ACGACCACTTTGTCAAGCTC
hGAPDH R		GTGAGGAGGGGAGATTCAGT

### CT26 subcutaneous tumor model and treatment with oncolytic adenoviruses

To establish the murine CRC model, 2 × 10^6^ CT26 cells/100 µL were subcutaneously injected into 4–6 weeks-old BALB/c mice. Fourteen days after transplantation, tumor volumes were measured by using the following formula: tumor volume = Width^2^ × Length/2. Tumor-bearing mice were divided into five groups without statistical difference in tumor volume (*n* = 20/group). And, 2.5 × 10^10^ VPs/100 µL rAd.mDCN.mCD40L, rAd.mDCN, rAd.mCD40L, rAd.Null, or 100 µL phosphate-buffered saline (PBS) was administrated intratumorally on day 0. On day 3, a repeated injection was conducted. The heathy and survival of mice in each group were monitored every day. And, tumor volumes were measured twice a week. All procedures of animal experiments were approved by the Committee on Animal Care and Use and the Committee on the Ethics of Animal Experiments of Ningbo University.

On day 7, five mice from each group were euthanized for analyzing the viral distribution, viral induced inflammatory responses and gene expression by histopathological analysis and real-time RT-PCR. On day 25, the survived mice were euthanized for analyzing tumor structure, tumor metastasis, and expression of inflammatory cytokines.

### Histopathological analysis

On days 7 and 25, tumor lesions, liver, spleen, and lung were harvested, processed, and stained with hematoxylin and eosin (H&E). Then, the distribution of oncolytic adenoviruses in the tumor was analyzed by immunohistochemistry (IHC) using a mouse anti-adenovirus antibody (Abcam, Cambridge, MA, USA), a goat anti-mouse decorin antibody (R&D Systems, MN, USA) and a rabbit anti-mouse CD40L antibody (Invitrogen, Carlsbad, CA, USA) on day 7. Moreover, the apoptosis in tumor lesions was examined on day 7 by terminal deoxynucleotidyl transferased UTP nick end labeling (TUNEL; Promega, Madison, WI, USA) according to the manufacturer’s instructions.

### Immunophenotype analysis of peripheral blood cells

On days 7, 14, 21 and 25, peripheral blood samples were collected from the orbital sinus, and the subtypes of T lymphocytes, including CD8^+^ effector T cells and CD4^+^ memory T cells, bone marrow derived suppressor cells (MDSCs) and regulatory T cells (Tregs) were analyzed by flow cytometry. Briefly, the blood samples were labelled with antibody panels for 30 min at room temperature. And then, the erythrocytes were lysed by red blood cell (RBC) lysis buffer (BD Biosciences, San Jose, CA, USA) for 10 min at room temperature. Finally, the immune phenotypes were analyzed by flow cytometry.

The antibody panels were listed as followed. CD8^+^ effector T cells: APC-conjugated hamster anti-mouse CD3e antibody (APC-CD3e), FITC-conjugated CD8a Monoclonal Antibody (53 − 6.7), PE-conjugated CD69 Monoclonal Antibody (H1.2F3); CD4^+^ memory T cells, FITC-conjugated CD4 Monoclonal Antibody (GK1.5), PE-conjugated CD44 Monoclonal Antibody (IM7), APC-conjugated anti-mouse CD62L Antibody; MDSCs, FITC-conjugated CD11b Monoclonal Antibody (M1/70) and PE-conjugated Rat Anti-Mouse Ly-6G and Ly-6 C. Tregs, FITC-conjugated CD4 Monoclonal Antibody (GK1.5), APC-conjugated CD25 Monoclonal Antibody (PC61.5), PE-conjugated FOXP3 Monoclonal Antibody (FJK-16s). All of the antibodies were purchased from eBioscience.

### Statistical analysis

Data are presented as mean ± s.e.m., and were statistically analyzed using GraphPad Prism v7 (GraphPad Software, San Diego, CA). Longitudinal data, such as tumor growth curve, were analyzed using two-way repeated measure ANOVA followed by Bonferroni post hoc tests. And, other data were analyzed by one-way ANOVA and followed by Bonferroni post hoc tests. The differences were considered as significant at two side *p* < 0.05.

## Data Availability

All data are available in the main text.
